# Abscisic Acid as Pathogen Effector and Immune Regulator

**DOI:** 10.3389/fpls.2017.00587

**Published:** 2017-04-19

**Authors:** Laurens Lievens, Jacob Pollier, Alain Goossens, Rudi Beyaert, Jens Staal

**Affiliations:** ^1^Unit of Molecular Signal Transduction in Inflammation, VIB-UGent Center for Inflammation Research, VIBGhent, Belgium; ^2^Department of Biomedical Molecular Biology, Ghent UniversityGhent, Belgium; ^3^VIB-UGent Center for Plant Systems Biology, VIBGhent, Belgium; ^4^Department of Plant Biotechnology and Bioinformatics, Ghent UniversityGhent, Belgium

**Keywords:** immunity, inflammation, host-microbe interactions, metabolic engineering, comparative biology, signal transduction, natural product chemistry, systems biology

## Abstract

Abscisic acid (ABA) is a sesquiterpene signaling molecule produced in all kingdoms of life. To date, the best known functions of ABA are derived from its role as a major phytohormone in plant abiotic stress resistance. Different organisms have developed different biosynthesis and signal transduction pathways related to ABA. Despite this, there are also intriguing common themes where ABA often suppresses host immune responses and is utilized by pathogens as an effector molecule. ABA also seems to play an important role in compatible mutualistic interactions such as mycorrhiza and rhizosphere bacteria with plants, and possibly also the animal gut microbiome. The frequent use of ABA in inter-species communication could be a possible reason for the wide distribution and re-invention of ABA as a signaling molecule in different organisms. In humans and animal models, it has been shown that ABA treatment or nutrient-derived ABA is beneficial in inflammatory diseases like colitis and type 2 diabetes, which confer potential to ABA as an interesting nutraceutical or pharmacognostic drug. The anti-inflammatory activity, cellular metabolic reprogramming, and other beneficial physiological and psychological effects of ABA treatment in humans and animal models has sparked an interest in this molecule and its signaling pathway as a novel pharmacological target. In contrast to plants, however, very little is known about the ABA biosynthesis and signaling in other organisms. Genes, tools and knowledge about ABA from plant sciences and studies of phytopathogenic fungi might benefit biomedical studies on the physiological role of endogenously generated ABA in humans.

## Introduction

Abscisic acid (ABA) is best known as a phytohormone regulating abiotic stress responses in plants, but ABA biosynthesis has been observed in a phylogenetically wide range of organisms (Hartung, 2010), ranging from cultured cyanobacteria (Maršálek et al., 1992) to human cells (Bruzzone et al., 2007). Although commonalities in ABA response between cells from diverse organism classes have been observed (Huddart et al., 1986), this evolutionary ancient signaling molecule shows several kingdom-specific features in both biosynthesis and signaling (Hirai et al., 2000; Hauser et al., 2011). It is not known why this particular molecule is produced by so many different types of organisms and why so many different types of organisms respond to ABA. Furthermore, ABA can exist in four different possible stereoisomeric forms, but naturally occurring ABA is predominantly of a single form [(+)*cis, trans*-ABA]. There are no special chemical properties that make ABA particularly useful as a signaling molecule, compared to all other potential alternative chemical structures. Despite the kingdom-specific signaling and biosynthesis, there are also commonalities between plants and animals where both plants and animals rely on intracellular free ABA for activation of their receptors (Klingler et al., 2010; Sturla et al., 2011). Intracellular ABA homeostasis is regulated by several different mechanisms: biosynthesis/catabolism, conjugation/deconjugation and export/import (Dong et al., 2015). Many of the mechanisms regulating intracellular free ABA homeostasis are however currently only known in plants. The vast majority of all studies on physiological effects of ABA to date are also focused on the role of ABA in plants. Most notably among studies of ABA in other organisms, several phytopathogenic fungal pathogens produce ABA as a virulence factor (Mbengue et al., 2016), and some mutualistic host-microbe interactions also rely on ABA (Stec et al., 2016). ABA influences immune responses in animals, and also animal pathogens can utilize this molecule as an effector molecule (Wang et al., 2009). Apart from direct effects on immunity, ABA also seems to influence metabolic control which might play a role in its protective activity against diabetes. In addition to endogenous biosynthesis, humans and other animals will also have a constant exposure of ABA from nutritional sources, and there are indications that a high ABA diet can have beneficial physiological effects (Magnone et al., 2015). One possible reason why ABA is so widely distributed among different types of organisms and why biosynthesis, perception and signaling for this molecule has been re-invented several times could be that ABA often is used as a signaling molecule for communication between different species. If that is the case, the role of ABA in host-pathogen and mutualistic interactions would be ancient and a universal theme among different organisms and organism interactions. The physiological effects of ABA in animals, especially related to immunity, inflammation and metabolic control, have sparked an interest in utilizing this pathway as a pharmacological target (Sakthivel et al., 2016), which has led to several recent papers on ABA functions in animals. However, the most famous plant hormone with pharmacological effects in humans is salicylic acid (SA). Just like with ABA, humans and other animals are constantly exposed to low doses of SA as part of their diet, which can have physiological effects (Klessig et al., 2016). Despite the well-documented effects of SA both in plants and animals, it is not until recently that several molecular targets have been identified (Manohar et al., 2014; Choi et al., 2015a,b, 2016; Dachineni et al., 2016). Earlier molecular targets of SA were often either only active at too high concentrations to be physiologically relevant, or only inhibited by acetyl-SA (i.e., Aspirin vs. COX-1 and -2). Interestingly, some of these new molecular targets are common between plants and animals (e.g., HMGB1 in humans and related plant homologs), and there are some indications of an endogenous SA biosynthesis pathway in humans (Klessig et al., 2016). This near-universal use of certain molecular structures in many different organisms and organism interactions, together with their interesting nutritional and pharmacological effects calls for a highly multidisciplinary approach in order to further elucidate the role and functions of ABA and other plant hormones in non-plant organisms. Here we will discuss what is known about the alternative “direct” biosynthesis pathway of ABA in fungi, and the role of ABA in immunity and inter-species communication. We will also explore the possibilities to utilize genes and tools from plants and phytopathogenic fungi for functional evaluation of the role of ABA in animals.

## Different ABA biosynthesis pathways

**Figure 1:**
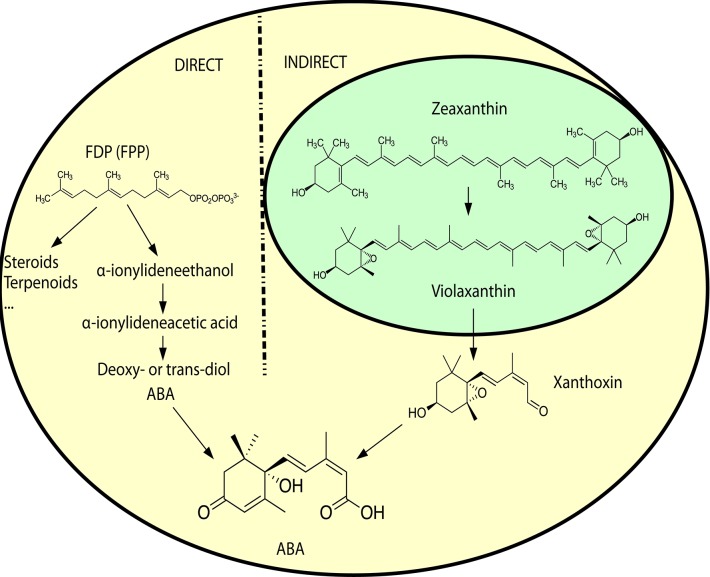
**A simplified overview of the direct ABA biosynthesis in fungi and the indirect biosynthesis in plants**. Yellow background indicates cytosolic compartment, and green background the chloroplast compartment. For more detailed biosynthetic pathways, see Siewers et al. (2006) for the direct pathway in fungi and Finkelstein (2013) for the indirect pathway in plants. Molecular structures in scalable vector graphics (SVG) format were obtained from Wikipedia (https://en.wikipedia.org) on 2017-02-08, and the images were licensed “public domain” by the original authors.

**Figure 2:**
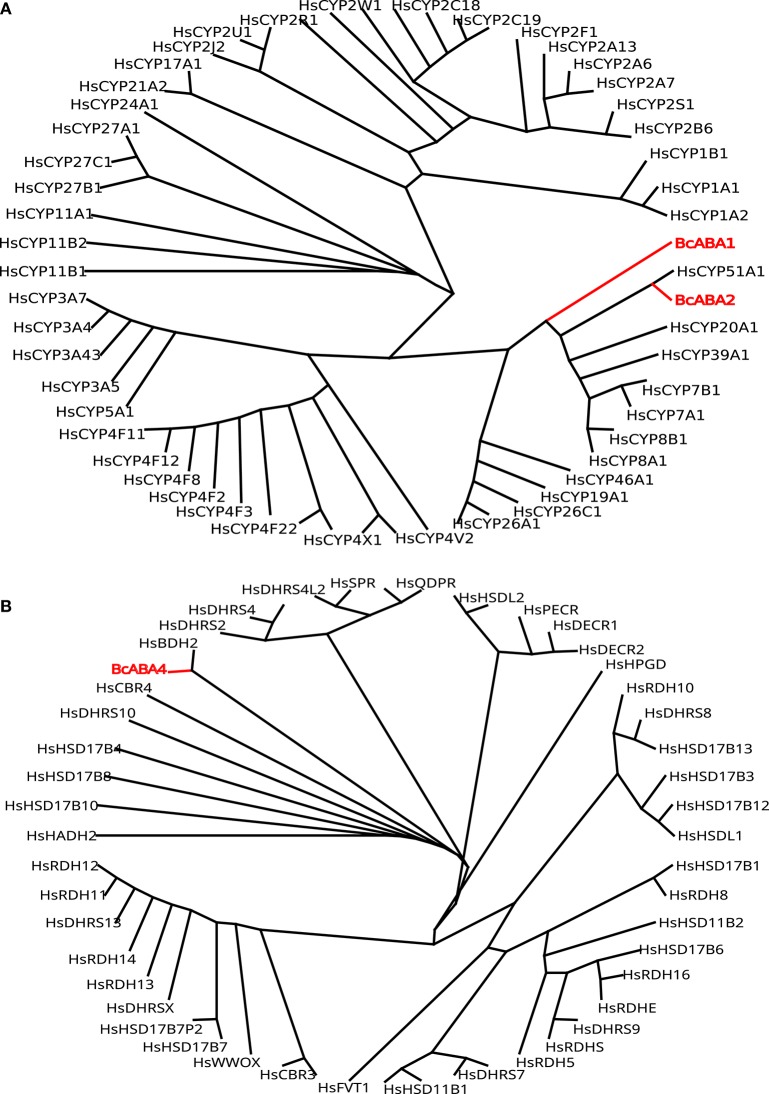
**(A)** Phylogeny of *Botrytis* P450 ABA biosynthesis proteins BcABA1 and BcABA2 (red) compared to all human P450 proteins (black). **(B)** Phylogeny of *Botrytis* BcABA4 biosynthesis protein (red) compared to all human carbonyl reductase family members (black).

ABA is a sesquiterpene (15 carbons) that can be found in many different kinds of organisms which produce it by different means (Hauser et al., 2011) (Figure 1). Plants synthesize their ABA via their plastids (an organelle that does not exist in fungi or animals) as a compound derived from large (40 carbons) carotenoid precursor molecules generated via the plastidial 2-*C*-methyl-D-erythritol 4-phosphate (MEP) pathway (Schwartz et al., 2003; Finkelstein, 2013). Rhizosphere and endophytic bacteria, such as *Achromobacter, Bacillus* and *Pseudomonas*, have all been shown to produce ABA in axenic cultures (Forchetti et al., 2007; Salomon et al., 2014). Also marine *Streptomyces* isolates have been shown to produce ABA and several other phytohormones without any obvious interaction with plants (Rashad et al., 2015). No ABA biosynthesis pathway is currently described in ABA-producing bacteria, but *Achromobacter, Bacillus* and *Pseudomonas* are all known to produce carotenoids, which makes it likely that also these bacteria depend on a carotenoid-dependent pathway for generation of ABA. Further underscoring this, in plants ABA synthesis from carotenoids takes place in the plastids, cell organelles thought to have originated from endosymbiotic cyanobacteria. It is however possible that different prokaryotic species utilize independently evolved carotenoid-dependent and -independent ABA biosynthesis pathways. Several phytopathogenic fungi, such as *Cercospora rosicola* (Assante et al., 1977), *Botrytis cinerea* (Hirai et al., 1986) and *Magnaporthe oryzae* (Spence et al., 2015) have been shown to produce ABA. In contrast to the plant carotenoid-dependent “indirect” pathway, the fungal ABA biosynthesis depends on a “direct” pathway with a 15 carbon (farnesyl diphosphate (FDP) or farnesyl pyrophosphate (FPP)) precursor molecule generated via the mevalonate (MVA) pathway (Hirai et al., 2000). FDP is a common and often rate-limiting precursor for several metabolites synthesized through the MVA pathway, including steroids, also in animals (Park et al., 2017). Several genes critical for this “direct” ABA biosynthesis have been identified in the broad host range phytopathogenic gray mold fungus *B. cinerea*: the P450 reductase CPR1 (Siewers et al., 2004) and a biosynthesis gene cluster (BcABA1-4) (Siewers et al., 2006). The enzyme catalyzing the first committed step in the fungal ABA biosynthesis pathway, the conversion of FDP to allofarnesene and ionylideneethane, is however not yet known (Siewers et al., 2006). Genetic and transcriptional studies of ABA over-expressing strains of *B. cinerea* (Ding et al., 2016) will hopefully result in the identification of genes for missing critical metabolic steps and a more complete general understanding of the direct ABA biosynthesis pathway in various phytopathogenic fungi. As a complementary strategy, defining the phylogenetic distribution of fungal ABA biosynthesis could further help to define and identify the genes responsible for critical biosynthetic steps. Other fungal pathogens, such as the rice blast pathogen *M. oryzae*, rely on the same biosynthesis genes as in *B. cinerea* (Spence et al., 2015). Furthermore, an NCBI BLASTp (Johnson et al., 2008) survey of homologs of the protein sequences from the *B. cinerea* ABA biosynthesis cluster easily identifies closely related proteins in many different endophytic or pathogenic fungi (e.g., *Alternaria, Aspergillus, Aureobasidium, Colletotrichum, Dothistroma, Eutypa, Fusarium, Leptosphaeria, Magnaporthe, Pyrenophora*, and *Verticillium*) and ABA has already been found in *Aspergillus, Fusarium* and many other kinds of saprophytic or parasitic fungi (Crocoll et al., 1991; Xu et al., 2008; Dörffling et al., 2014; Morrison et al., 2015; Uzor et al., 2017). Taken together, this might indicate that there is a conserved direct ABA biosynthesis pathway in fungi. It also indicates that ABA as a fungal virulence or compatibility strategy can be more common than currently appreciated. Fungi are part of the Opisthokont group together with animals (Steenkamp et al., 2006), which makes them closer related to animals than plants are and it is thus more likely that fungi and animals share a common ABA biosynthesis pathway. Of the four known genes in the *B. cinerea* ABA biosynthesis cluster, three (BcABA1, 2, and 4) have several homologs in the mammalian genomes and are thus candidate endogenous biosynthesis genes (Figure 2; Supplemental Texts 1, 2). In BLASTp surveys where fungi are excluded, animal homologs of BcABA2 are the top hits. BcABA3 homologs have a much narrower phylogenetic distribution (fungi, *Amycolatopsis* and *Streptomyces*), and this protein plays an unknown but critical biochemical role in *B. cinerea* ABA biosynthesis (Siewers et al., 2006). In contrast to what was previously reported (Spence et al., 2015), there is an *M. oryzae* BcABA3 homolog (Genbank: ELQ43177.1). Considering the long evolutionary distances between fungi and metazoa, it is possible that a conserved metabolic pathway will rely on unrelated proteins filling the same functions in animals and fungi through convergent evolution. Two of the critical ABA biosynthesis steps in *Botrytis* (BcABA1 and BcABA2) are represented by P450 proteins, and only CYP51 and CYP7 proteins are found in both fungi and metazoa (Nelson et al., 2013). It is however also possible that animals do not have a conserved biosynthetic pathway in common with fungi, and that they rely on carotenoid metabolites from nutritional sources to generate ABA in a manner similar to the retinoic acid biosynthesis pathway. Considering that humans lack the MEP pathway and that human cells are able to produce ABA in culture (Bruzzone et al., 2007), it is however more likely that animals rely on an endogenous “direct” (MVA) biosynthesis pathway similar to that of fungi. To distinguish between these two possibilities, it would be interesting to investigate this further in animal cells, for instance through isotope labeled metabolites, as previously done for fungi and plants (Hirai et al., 2000). Alternatively could ABA production from animal cells be investigated in presence of different types of inhibitors, such as statins and bisphosphonates, in order to determine the biosynthesis pathway.

## ABA in plant-microbe interactions

**Figure 3:**
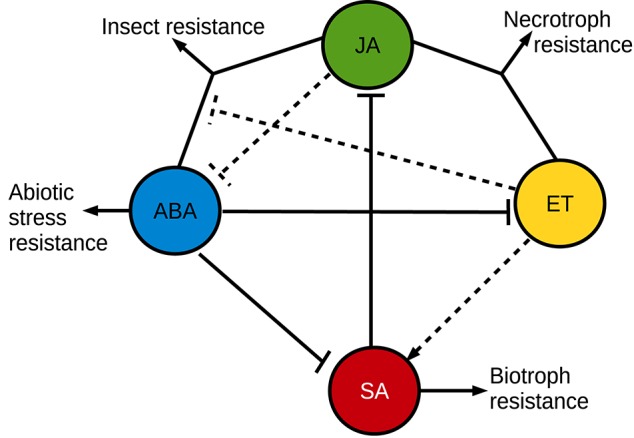
**A simplified overview of synergistic and antagonistic interactions between plant stress resistance hormone signaling pathways**. The simplified overview does not accurately describe the plant hormone involvement in all plant pathosystems, where variations may occur.

The effect of ABA on plant pathogen resistance is complex (Figure 3). Many pathosystems have demonstrated a negative effect from ABA on plant pathogen resistance, such as the *Botrytis cinerea*—tomato (Audenaert et al., 2002; Sivakumaran et al., 2016), *Ralstonia solanacearum*—tobacco (Zhou et al., 2008), *Plectosphaerella cucumerina*—*Arabidopsis* (Hernández-Blanco et al., 2007; Sánchez-Vallet et al., 2012), and *Magnaporthe oryzae*—barley (Ulferts et al., 2015) pathosystems. Emerging evidence also indicate that ABA plays an important role in compatible interactions in mutualistic plant-microbe interactions (Stec et al., 2016). For example, ABA is promoting infection and establishment of compatible interactions with arbuscular mycorrhizal fungi (Herrera-Medina et al., 2007; Charpentier et al., 2014; Fracetto et al., 2017) and several kinds of root-associated bacteria produce ABA in the rhizosphere (Salomon et al., 2014). Not only does ABA suppress immune responses, but immune responses are also able to suppress ABA signaling. For example can very early pathogen response signaling negatively regulate ABA responses (Kim et al., 2011; Desclos-Theveniau et al., 2012). In many diverse pathosystems, ABA acts antagonistically against the SA pathogen disease resistance hormone pathway (Audenaert et al., 2002; Jiang et al., 2010). Plants need to prioritize between many different environmental cues for an appropriate response, which could explain some of the antagonistic effects between different signaling pathways (Denancé et al., 2013; Vos et al., 2015; Kissoudis et al., 2016). The pathogen resistance responses can also be complex and multi-layered, where the effects of lesser responses only are apparent when the major responses have been disabled (Persson et al., 2009). Lately, complex interactions between multiple plant hormones and the traditional pathogen disease hormone [SA, jasmonic acid (JA) and ethylene (ET)] signaling pathways has been unraveled, which further highlights this inherent conflict between different plant responses (Shigenaga and Argueso, 2016; Verma et al., 2016). Also the different defense hormones play different mutually exclusive roles. In a simplified generalization, SA is involved in resistance against biotrophic pathogens and JA is involved in resistance against necrotrophic pathogens or insects. A vast majority of the studies of hormone interactions in disease resistance rely on the *Arabidopsis* model system, and there are indications that other plants may respond differently (De Vleesschauwer et al., 2014). As part of the antagonism between resistance against biotrophs and necrotrophs, SA suppresses JA responses at multiple levels (Caarls et al., 2015). ET, on the other hand, can enhance or influence both SA and JA responses (Broekgaarden et al., 2015). While the antagonism between ABA and SA is relatively clear in many models (de Torres Zabala et al., 2009; Moeder et al., 2010), the ABA influence on JA signaling is more complex where the JA/ABA branch regulates a different set of JA responses compared to the JA/ET branch, and these two branches are mutually exclusive (Anderson et al., 2004). The JA/ET branch responses rely on the ERF transcription factors, leading to expression of defensins and resistance against necrotrophs (Müller and Munné-Bosch, 2015). The JA/ABA branch responses, on the other hand, rely on the transcription factor MYC2, which regulates wounding responses, insect resistance and suppression of JA/ET-dependent innate immunity against necrotrophs (Kazan and Manners, 2013; Goossens et al., 2016). Interestingly, the transcription profile of genes downstream of MYC2 might be influenced directly by ABA via physical interactions with one of the intracellular ABA receptors (Aleman et al., 2016). In addition to the immune suppressive effects from ABA, it can also play a positive role in pathogen resistance. One of the first indications of a positive influence from plant ABA signaling on biotic stress resistance was the reliance of ABA for a β-aminobutyric acid (BABA)-induced priming for pathogen resistance (Ton and Mauch-Mani, 2004). Endogenous ABA was later shown to play a positive role directly in *Brassica napus* and *Arabidopsis* callose-dependent disease resistance to the hemibiotrophic fungal pathogen *Leptosphaeria maculans* (Kaliff et al., 2007). ABA is often a positive regulator of the callose-dependent disease resistance responses (Ton and Mauch-Mani, 2004; Flors et al., 2008; García-Andrade et al., 2011; Oide et al., 2013). Callose is a rapidly formed β-glucan barrier, which in turn also is in conflict with SA-dependent disease resistance responses (Nishimura et al., 2003; Oide et al., 2013). Also other kinds of physical barriers seem to interact with ABA and influence plant disease resistance, for example are *Arabidopsis* ABA-dependent resistance responses against *R. solanacearum* constituitively up-regulated in certain cellulose synthase mutants (Hernández-Blanco et al., 2007; Feng et al., 2012). In a more classical signaling sense, ABA has also been found to play a positive role together with JA in the resistance to *Sclerotinia sclerotiorum* in *Arabidopsis* (Perchepied et al., 2010), and the JA/ABA pathway has subsequently also been identified as important for resistance against insects (Verhage et al., 2011; Vos et al., 2013). An antagonistic action of ABA on ET-dependent infection has also been suggested as a mechanism in rice resistance against *Cochliobolus miyabeanus* (De Vleesschauwer et al., 2010). That ET can act as a virulence-promoting signal has been seen also in other pathosystems where ABA plays a positive role in resistance (Persson et al., 2009; Groen et al., 2013). In contrast, the ABA-ET antagonism is also important for establishment of arbuscular mycorrhiza where ET suppresses succesful colonization in ABA-deficient plants, most likely due to activation of JA/ET-dependent disease resistance responses against the invading fungus (Garrido et al., 2010; Fracetto et al., 2017). As an alternative mechanism, ABA can also positively influence disease resistance by regulating stomatal closure in order to deny pathogens entry into the plant (Lim et al., 2015). Interestingly, the JA-dependent pathway can sometimes antagonize this ABA-induced stomatal closure, which provides another example of opposing effects from ABA and other plant disease resistance hormones. Pathogens such as *Pseudomonas syringae* and various fungi can “hijack” the inherent antagonism between different resistance pathways in order to promote infection by using plant hormones or plant hormone-like compounds as effector molecules (Goossens et al., 2016; Toum et al., 2016).

## ABA as pathogen effector in plant and animal hosts

Several phytopathogenic fungal pathogens produce ABA and other small molecule effectors in order to suppress plant immune responses (Mbengue et al., 2016), and there are indications that also human pathogens use ABA for a similar strategy (Wang et al., 2009). Some pathogens, like *P. syringae*, indirectly utilizes ABA as an effector molecule by modulating the endogenous ABA biosynthesis and response pathways in the host plant (de Torres-Zabala et al., 2007), which in turn indirectly modulates mutually exclusive pathogen responses, like the antagonistic relationship between SA- and callose-dependent responses (Oide et al., 2013). Other pathogens have their own ABA biosynthesis, most notably several phytopathogenic fungi such as *B. cinerea*. Most studies on *B. cinerea* ABA biosynthesis focus on its production in fermentation (Ding et al., 2015, 2016), and there are unfortunately no studies that have established the effect of altered *B. cinerea* ABA biosynthesis on virulence. The rice blast fungus *M. oryzae*, which depends on the same ABA biosynthesis pathway as *B. cinerea* (Spence et al., 2015), rely on ABA for virulence and respond to ABA in order to form appressoria for infection. The plastid-containing protozoan animal parasite *Toxoplasma gondii* also utilizes ABA as a virulence factor to suppress animal host responses by inducing autophagy (Wang et al., 2009). In contrast, the related malaria parasite *Plasmodium* produces SA to suppress animal host immunity, and ABA treatment seems to reduce the virulence of this pathogen (Matsubara et al., 2015; Glennon et al., 2016). Interpretations of the role of ABA in *Toxoplasma* virulence must however be made with caution, since *Toxoplasma* ABA production was inhibited using the herbicide Fluridone which also seems to affect ABA produced in mammalian cells (Magnone et al., 2013). Because of this, it would be interesting with transgenic ABA depletion studies by overexpression of an ABA catabolic enzyme in *Toxoplasma*, similar to how SA was depleted in *Plasmodium* by overexpression of a bacterial salicylate hydroxylase (Matsubara et al., 2015). Other pathogens, like the oomycete oyster parasite *Perkinsus marinus*, also produce ABA but it does not seem to influence its virulence (Sakamoto et al., 2016). ABA can also directly influence pathogen growth and development, such as the rice blast fungus *M. oryzae* (Spence et al., 2015) or the protist animal parasite *T. gondii* (Nagamune et al., 2008). In this context, the effect of ABA on pathogen development can fit into a much broader observation of complex hormone interactions between the host and the virulence of certain pathogens, like *T. gondii* (Galván-Ramírez et al., 2014). The use of ABA and other plant hormones as effector molecules or virulence factors on non-plant hosts is still a relatively unexplored area, and it is possible that more bacterial, protist, oomycete and fungal pathogens utilize this strategy.

## Physiological and pharmacological effects of ABA in animals

The presence of ABA in animal tissues has been known since the 1980s (Le Page-Degivry et al., 1986; Li et al., 2011), but has largely been ignored until recently. In early branching metazoans, ABA has been associated to abiotic stress responses (Zocchi et al., 2001) and nutrient-derived ABA has been suggested to promote innate immunity in honey bees (Negri et al., 2015). Administration of very high doses of ABA is well tolerated in mice without adverse effects (Li et al., 2011), which makes ABA treatments pharmacologically interesting. There are however reports that long-term ABA exposure might have adverse effects (Isik and Celik, 2015), which calls for caution. ABA treatment in humans and animal models has been suggested to be beneficial for type 2 diabetes (Guri et al., 2007; Bruzzone et al., 2015; Magnone et al., 2015), inflammatory bowel disease (IBD) (Guri et al., 2011; Viladomiu et al., 2013), atherosclerosis (Guri et al., 2010), systemic sclerosis (Bruzzone et al., 2012c), glioma (Zhou et al., 2016), depression (Qi et al., 2015b, 2016), and resistance against hepatitis C (Rakic et al., 2006), influenza (Hontecillas et al., 2013), malaria (Glennon et al., 2016), and mycobacteria (Clark et al., 2013). In addition to being administered either through nutritional sources or as a drug, there is also endogenous production of ABA in many different types of mammalian cells—such as stem cells, macrophages, microglia, granulocytes and keratinocytes (Bruzzone et al., 2007, 2012b; Scarfì et al., 2008; Bodrato et al., 2009; Magnone et al., 2009, 2012). Furthermore, endogenous induction of serum ABA in response to glucose challenge has been shown to stimulate insulin release and is impaired in patients suffering from type 2 diabetes (Bruzzone et al., 2008, 2012a; Ameri et al., 2015). ABA has recently attracted the attention of several research groups as a potential drug lead (Bassaganya-Riera et al., 2010; Sakthivel et al., 2016) because of these promising biological activities. One study has already shown that fruits and vegetables with high ABA contents (such as figs and apricots) are beneficial against hyperglycemia in both rats and humans (Magnone et al., 2015), which makes ABA a potential pharmacognostic drug or nutraceutical. Pharmaceutical research on ABA currently focuses on generation of ABA analogs that will be more stable or act as ABA antagonists (Grozio et al., 2011; Bellotti et al., 2015). Similar approaches are currently also pursued in the plant sciences where antagonistic ABA analogs have been designed (Takeuchi et al., 2014), and pyrabactin variants are utilized as more stable and chemically unrelated ABA agonists (Overtveldt et al., 2015). An alternative treatment approach is to manipulate ABA homeostasis indirectly by targeting the different mechanisms that influence the levels of free intracellular ABA (Todoroki and Ueno, 2010). A conceptually similar approach has already been tried in animal systems by treatments with a herbicide (Fluridone), which is known to influence ABA levels in plants (Magnone et al., 2013). At this moment, there are no reports describing an inhibition of the “direct” fungal ABA biosynthesis by Fluridone. How Fluoridone would influence ABA in mammalian cells is also unclear since the molecular target is the carotenoid biosynthesis protein phytoene desaturase (Chamovitz et al., 1993), which is absent in animals. More knowledge of the ABA homeostasis in humans and the molecular target of the herbicide is thus needed before it can be applied as a true drug strategy. There are however conflicting reports whether ABA plays a pro- or anti-inflammatory role in specific cell models (De Flora et al., 2014), but the over all pharmacological properties are anti-inflammatory.

**Figure 4:**
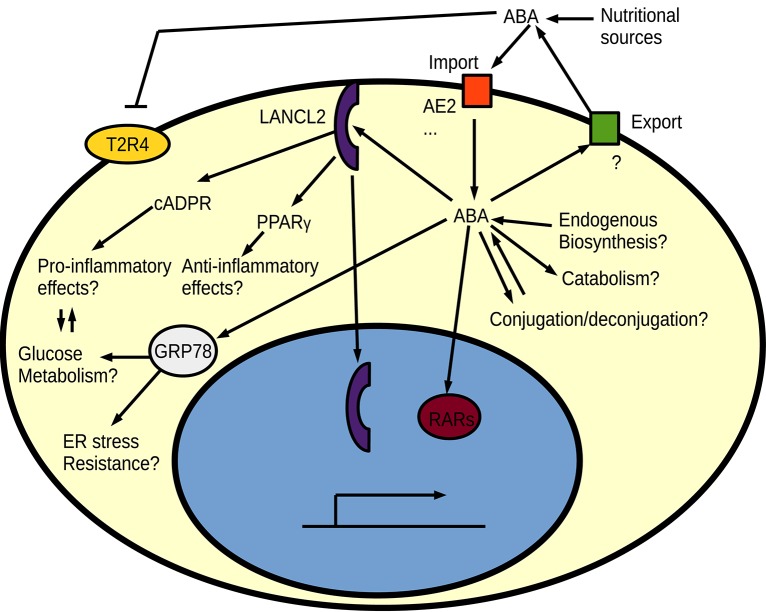
**An overview of proposed signaling pathways for ABA in animal cells**. Regulation of intracellular free ABA homeostasis by biosynthesis/catabolism, export/import and conjugation/deconjugation are currently unknown in animals. Different ABA receptors have been proposed for ABA, such as the bitter taste receptor (T2R4), LANCL2, retinoic acid receptors (RARs), and HSP70 (GRP78).

The best studied proposed receptor for ABA in animals is the intracellular protein LANCL2, which is attached to the cell membrane by myristoylation (Sturla et al., 2009; Fresia et al., 2016). LANCL2 is homologous to the earlier proposed ABA receptor in plants: GCR2 (Liu et al., 2007; Chen and Ellis, 2008) and bacterial LanC lanthionine synthesis proteins. However, the mammalian LanC-like homologs do not show any evidence of having a conserved role in lanthionine biosynthesis (He et al., 2017). It is not yet known whether a currently unknown catalytic activity of LANCL2 could contribute to ABA signaling. GCR2 was proposed as a G-protein coupled receptor (GPCR) for ABA, but this was later challenged (Gao et al., 2007; Chen and Ellis, 2008) and an entirely different protein family (PYR/PYL/RCAR) of intracellular receptors is now thought to be the dominant ABA receptors in plants (Ma et al., 2009; Miyazono et al., 2009; Park et al., 2009). Signaling of ABA via the PYR/PYL/RCAR intracellular receptor family and its associated PP2C phosphatases is however a land plant-specific innovation with no animal homologs (Hauser et al., 2011). Despite the apparent low importance of GCR2 in plant ABA responses, the human LANCL2 seem to work as expected from an ABA receptor, since independent drugs targeting this protein result in effects similar to those seen by ABA treatment (Bissel et al., 2016). ABA is suggested to act through a LANCL2-PPARγ axis for its anti-inflammatory functions (Guri et al., 2008; Sturla et al., 2009, 2011) and through a GPCR—like signaling for pro-inflammatory responses (Bruzzone et al., 2007, 2012b; Sturla et al., 2009; Fresia et al., 2016). Interestingly, ABA worsened the inflammation in models of IBD when PPARγ was absent from T cells (Guri et al., 2011; Viladomiu et al., 2013), indicating that ABA indeed plays a dual role and acts both pro- and anti-inflammatory. Apart from its inflammation-modulating functions, ABA signals via LANCL2 have also been linked to metabolic reprogramming of adipocytes into brown fat cells (Sturla et al., 2017), which can be especially beneficial in the context of diabetes where a dual direct effect on immune cells and metabolism could contribute to reduced inflammation (Ray et al., 2016). One possible mechanism for the metabolic reprogramming is an effect of LANCL2 on the Akt/mTORC2 pathway, where LANCL2 influences insulin-dependent Akt phosphorylation (Zeng et al., 2014). The metabolic reprogramming and inflammation-modulating effects of ABA can however also be two sides of the same coin, since there is significant cross talk between inflammatory signaling pathways and metabolism in innate immune cells (Kelly and O'Neill, 2015). An increased oxidative metabolism through ABA signaling should promote the alternatively activated and often anti-inflammatory macrophages. Inspired by the protective effects of ABA, LANCL2 has been proposed as a drug target, especially for treatments of IBD (Lu et al., 2011, 2014; Basson et al., 2016). Apart from the proposed GPCR-like signaling and the PPARγ mediated signaling, LANCL2 has some interesting potential direct signaling effects. For example, binding of ABA causes nuclear translocation of LANCL2 (Fresia et al., 2016), and nuclear LANCL2 has already been shown to influence transcription factor activity (Park and James, 2003). On the other hand are there no spontaneous phenotypes reported for LANCL2 deficient mice (Leber et al., 2016). The lack of spontaneous phenotype in *LancL2* knock-out mice is also not due to redundancy with the other mammalian LanC-like proteins, since the *LancL1-3* triple knock-out mice still are viable and do not show any gross abnormalities (He et al., 2017). The downstream PPARγ is known to be required for alternative (M2) activation of macrophages (Odegaard et al., 2007). Macrophages can be activated (polarized) in two different ways: the classically activated pro-inflammatory M1 macrophages, and different kinds of alternatively activated M2 macrophages that often act anti-inflammatory and are important for tissue repair (Mantovani et al., 2002). The PPARγ–mediated M2 polarization could explain the anti-inflammatory role of ABA. Consistent with the proposed LANCL2-PPARγ ABA signaling pathway, *LancL2* full-body or myeloid-specific knock-out mice show an impaired development of regulatory anti-inflammatory macrophages in response to *Helicobacter pylori* infection, which results in a stronger immune response but also greater tissue damage (Leber et al., 2016). It will be important to evaluate these *LancL2* knock-out mice or the triple knock-out mice for ABA responses in models that have already shown clear effects from ABA—including models where other ABA receptors have been proposed. One such alternative pathway suggests that ABA acts as an agonist to the retinoic acid nuclear receptors (Zhou et al., 2016). ABA stimulation through cross-reactivity with the retinoic acid receptors could also cause anti-inflammatory effects, since retinoic acid has been shown to be protective in inflammatory models of rheumatoid arthritis (Kwok et al., 2012). Surveys of other ABA-binding human proteins have also identified HSP70 family members as ABA-binding (Kharenko et al., 2013), and ABA-mediated megakarocyte survival has been suggested to rely on both LANCL2 and HSP70 (GRP78) interactions with ABA (Malara et al., 2017). The possible role of HSP70 family members in ABA signaling is another intriguing parallel with plant ABA signaling, where overexpression of HSC70 caused an ABA-hypersensitive phenotype in *Arabidopsis* (Clément et al., 2011). ABA seems to bind many potential receptors (Figure 4) and seems to perform several physiological functions (Scarfì et al., 2009; Wang et al., 2009; Kharenko et al., 2013; Pydi et al., 2015; Qi et al., 2015a). Whether LANCL2 is the major ABA receptor in humans is thus still not entirely certain.

An intriguing indirect alternative effect of ABA is its role in inhibiting the bitter taste receptor, which is known to influence the gut microbiome and glucose responses (Dotson et al., 2008; Pydi et al., 2015; Latorre et al., 2016). This could explain many physiological effects of ABA, especially its protective effects in colitis and diabetes, and possibly also some psychological effects via the gut-brain axis. Gut bitter taste receptors would also be located at a primary site for nutrient-derived ABA, but it can also mean that microbe-generated ABA is an important signal between the gut microbiome and the host since gut microbes produce many complex metabolites (Lee and Hase, 2014; Palau-Rodriguez et al., 2015). Cultures of bacteria found in the animal gut (*Escherichia coli, Klebsiella pneumonia* and *Proteus mirabilis*) can produce low levels of ABA and other plant hormones (Karadeniz et al., 2006), but there are currently no reports on whether ABA or other plant hormones are present in the endogenous gut microbiome metabolome and if levels and presence of these compounds correlate with different host physiological states.

Being an endogenously generated anti-inflammatory secondary metabolite puts ABA in the same category as the glucocorticoids and other anti-inflammatory steroids, but with the important difference that we know very little about the function and regulation of ABA in humans. It is possible that the mammalian ABA receptor(s) are equally elusive as they used to be in plants prior to the discovery of the PYR/PYL/RCAR receptor family, with several ABA-binding proteins being proposed as receptor candidates (Klingler et al., 2010). It is also possible that many molecular targets of ABA will hit a gray zone where the question whether they should be considered receptors turns into a matter of semantics, similar to the current situation with SA (Klessig et al., 2016). It is however likely that there will be analogies between plant and animal ABA receptors. Intracellular receptors are for example likely, and since ABA cannot pass the cell membrane at physiological pH (Kuromori and Shinozaki, 2010), active transport is needed for signaling. Recently, two different ABA importers were identified in mammals (Vigliarolo et al., 2015, 2016), and this family of importers will most likely play an important role in regulating the ABA responsiveness of different cells. There could also be different receptor- and signal systems to respond to intracellular and extracellular ABA, which perhaps could explain some discrepancies between results suggesting an anti-inflammatory role of ABA and results where depletion of extracellular ABA with antibodies resulted in reduced activation of macrophages (Magnone et al., 2012). It is however also possible that ABA, just like in plants, plays a complex role in animal immunity where some immune responses are promoted whereas others are inhibited.

## Perspectives: how plant science and studies of phytopathogenic fungi can help biomedical science

The near universal presence of ABA and its intriguing physiological effects in different organisms calls for a highly multidisciplinary approach to further understand its endogenous functions in the different organisms as well as its pharmacological potential (Bohlin et al., 2010). Similar approaches might also be fruitful for studies of other signaling molecules from plants and phytopathogenic fungi that have been found to show pharmacological effects in animals, like for example gibberellins (Hedden and Thomas, 2012; Annand et al., 2015; Bannon et al., 2015; Reihill et al., 2016) and SA (Klessig et al., 2016). Since no genes are known that regulate intracellular ABA homeostasis in animals, utilizing proteins and pathways known in plants and phytopathogenic fungi could help defining the physiological role of ABA in animals. The plant P450 (CYP707A) enzymes that degrades ABA to phaseic acid (Umezawa et al., 2006) could be used to deplete endogenous ABA (or intracellular ABA from other sources) in transgenic animals or animal cells. CYP707As are active in presence of animal P450 reductases, but expression of the *Arabidopsis* P450 reductase ATR1 (At4g24520) results in 70% higher activity (Saito et al., 2004). A transgenic approach comparing at least 2 different inactivation pathways is also important, since metabolic engineering carries a risk of phenotypic artifacts from the degradation products (van Wees and Glazebrook, 2003). Recently, the CYP707A ABA degradation product phaseic acid was found to be active in both mammalian and plant cells, which could cause such phenotypic artifacts (Hou et al., 2016; Rodriguez, 2016; Weng et al., 2016). This means that future ABA depletion studies relying on CYP707A activity should most likely also express the phaseic acid reductase (PAR, *Arabidopsis ABH2:* At4g27250) to avoid one potential side effect. One alternative ABA inactivation pathway is a UDP-dependent glycosyl transferase (*UGT71C5, Arabidopsis*: At1g07240), which inactivates free ABA by conjugating it to glucose (Liu et al., 2015). However, overexpression of this enzyme in plants gives a much lower level of ABA insensitivity compared to CYP707A overexpressing plants. Other alternative ABA inactivation strategies are overexpression of intracellular small single chain antibody (scFv) against ABA (Genbank: Z29480.1) (Artsaenko et al., 1995) or overexpression of an ABA exporter (*ABCG25, Arabidopsis*: At1g71960) protein (Park et al., 2016). The general concept of translating tools from plant sciences to animal models could also be applicable for other signaling molecules. For example, since there are indications that there is an endogenous SA biosynthesis pathway and basal SA levels from nutrition in animals (Paterson et al., 2008; Klessig et al., 2016), a bacterial salicylate hydroxylase *nahG* (NCBI: NC_007926.1) transgenic (Gaffney et al., 1993; Matsubara et al., 2015) animal model to deplete endogenous SA (and intracellular SA from other sources) could give interesting insights on the physiological role of the basal levels of SA in animals. As with the ABA depletions, keeping in mind that also the SA degradation product catechol can have biological functions (van Wees and Glazebrook, 2003). Because of this, an alternative inactivation mechanism, like for example overexpression of SA carboxyl methyltransferase (AtBSMT1: At3g11480) (Liu et al., 2009), would be advisable as a complementary approach. Apart from ABA depletion by using plant proteins, it might also be possible to enhance ABA production in animal cells using genes from phytopathogenic fungi if the biosynthetic pathway is conserved (Ding et al., 2015). Plant science has also provided us with several methods to measure ABA. ELISA assays for ABA originally developed for plant science have already been used in many studies of ABA produced in animals. Unfortunately, the commercial kits are quite expensive which might act as a barrier to entry for many biomedical research groups that are not specifically working on ABA. The recently described production of recombinant small single chain (scFv) anti-ABA antibodies (Badescu et al., 2016) and the recent development of anti-ABA aptamers (Grozio et al., 2013) might however significantly simplify the generation of in-house ELISAs or lower the prices of commercial kits in the near future, which would make it a viable standard assay for clinical samples. Another very exciting novel development is the ability to measure intracellular free ABA concentrations in real-time using ABA FRET biosensors (Jones et al., 2014; Waadt et al., 2014). Expression of such biosensors in mammalian cells might help us to understand under which signaling conditions free intracellular ABA is produced in the cell, and might help us to more accurately investigate the regulation and dynamics of free intracellular ABA in mammalian cells.

## Author contributions

JS came up with the original ideas which form the foundation of this manuscript and wrote the manuscript. LL performed preliminary experiments in mammalian cells under guidance of JS and RB. JP, RB, and AG were involved in further planning, discussions and comments on the manuscript.

## Funding

Work in the Beyaert lab is financed by the Fund for Scientific Research Flanders (FWO), the Belgian Foundation Against Cancer, Interuniversity Attraction Poles, Concerted Research Actions (GOA) and the Group-ID Multidisciplinary Research Partnership of Ghent University.

### Conflict of interest statement

The authors declare that the research was conducted in the absence of any commercial or financial relationships that could be construed as a potential conflict of interest.

## Supplementary material

Supplemental Text 1 **FASTA sequences of *Botrytis* P450 BcABA1 and BcABA2 and all human P450 family member proteins**.

Supplemental Text 2 **FASTA sequences of *Botrytis* BcABA4 and all human carbonyl reductase family member proteins**.
